# Effects of Chronic Administration of a Standardized Rice Bran Supplement on Sleep Architecture and Profiles in Mice: A Comparative Study with a Hypnotic Drug Zolpidem

**DOI:** 10.4014/jmb.2505.05010

**Published:** 2025-08-26

**Authors:** Minseok Yoon, Sangoh Kwon, Min Young Um, Suengmok Cho

**Affiliations:** 1 Food Functionality Research Division, Korea Food Research Institute, Wanju 55364, Republic of Korea; 2 S&D Research and Development Institute, Cheongju 28156, Republic of Korea; 3 Department of Food Biotechnology, University of Science & Technology, Daejeon 34113, Republic of Korea; 4 Division of Food Science/Institute of Food Science, Pukyong National University, Busan 48513, Republic of Korea

**Keywords:** Rice, rice bran, hypnotic effect, chronic treatment, withdrawal

## Abstract

This study aimed to investigate the effects of chronic administration of rice bran supplement (RBS) on sleep and to compare these effects with those of the hypnotic drug zolpidem (ZPD). During the three-week treatment period, ZPD initially reduced sleep latency, but that reduction effect of sleep latency gradually diminished over time and significantly decreased non-rapid eye movement sleep (NREMS). In contrast, RBS reduced sleep latency and increased NREMS on the first day, and these effects persisted throughout the treatment period. Additionally, RBS altered the time course of NREMS, which persisted until day 21. Importantly, while RBS did not affect delta activity during the treatment period, the ZPD group experienced a decrease in delta activity on the first day, which recovered by day 21. Our findings suggest that RBS does not induce tolerance during chronic administration and does not alter sleep architecture upon discontinuation, indicating that it could be utilized as a natural sleep aid suitable for long-term consumption.

## Introduction

Insomnia is the most prevalent sleep disorder in modern industrialized societies, characterized by difficulties in initiating or maintaining sleep, or by early morning awakening. These symptoms often lead to daytime impairments such as fatigue, cognitive dysfunction, mood disturbances, and reduced occupational performance [1]. Chronic insomnia has been associated with a range of adverse health outcomes, including cardiovascular diseases, immune dysregulation, and impaired cognitive function, ultimately diminishing overall quality of life [2, 3].

In response to the limitations and side effects associated with conventional pharmacological treatments—such as tolerance, dependency, and withdrawal symptoms—there has been growing interest in natural sleep aids. Herbal remedies including valerian (*Valeriana officinalis*), hops (*Humulus lupulus*), and jujube (*Ziziphus jujuba*) have demonstrated sedative–hypnotic effects in both clinical and preclinical studies [4]. However, most existing research has focused primarily on the acute effects of these agents, with relatively few studies evaluating their efficacy and safety over prolonged use. Given that herbal supplements are often consumed continuously over long periods, chronic administration studies are essential to understand their sustained impact on sleep quality and potential side effects [5-7].

Rice bran, a byproduct of the rice milling process, has recently attracted attention as a potential functional food ingredient due to its rich content of bioactive compounds, including γ-oryzanol, vitamins, fatty acids, and phenolic compounds [8]. These compounds have been reported to exhibit antioxidant [9], anti-inflammatory [10], and neuroprotective [11] properties. In our previous studies, acute administration of a standardized rice bran supplement (RBS) was found to reduce sleep latency and promote non-rapid eye movement sleep (NREMS) in mice [12, 13]. However, since herbal sleep aids are often consumed over extended periods without the need for a prescription, it is essential to evaluate the effects of chronic administration in preclinical studies. Building upon these findings, the present study aimed to investigate the effects of chronic administration of RBS on sleep architecture in mice, with a specific focus on tolerance, sleep-stage modulation, and overall efficacy. Furthermore, we compared these effects with those of zolpidem (ZPD), a commonly prescribed hypnotic, to evaluate RBS as a potential natural alternative for long-term sleep support.

## Materials and Methods

### Materials

The RBS (lot no. SD-RB-002) was obtained from S&D Co., Ltd., (Republic of Korea). As described by the manufacturer, rice bran was extracted using an ethanol/water solution at 40°C for 8 h. The extraction solution was then filtered and concentrated. The concentrated extract, combined with d-α-tocopherol, polysorbate 20, sodium caseinate, and dextrin, was dried and powdered. RBS was standardized to contain 4.5 mg/g of γ-oryzanol. ZPD, a widely recognized GABA_A_-benzodiazepine receptor agonist (purchased from the Ministry of Food and Drug Safety, Republic of Korea), was selected as the reference hypnotic drug for comparison with RBS.

### Animals and Experimental Design

**Fig. 1:**
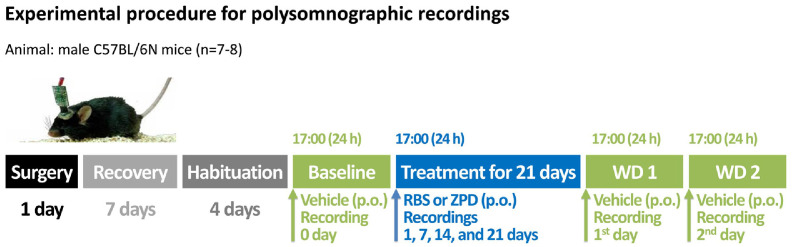
Experimental procedures and timeline for the polysomnographic recording. RBS, rice bran supplement; WD, withdrawal; ZPD, zolpidem.

All animal procedures in this study were approved by the Korea Food Research Institutional Animal Care and Use Committee (Approval number: KFRI-M-20021), and were performed in accordance with the institutional ethical guidelines. Male C57BL/6N mice (8-week-old, weighing 27–30 g) were obtained from Koatech Animal Inc., (Republic of Korea). The animals were housed in an insulated, soundproof recording room maintained at an ambient temperature of 23 ± 2°C and a relative humidity of 55 ± 2%, under an automatically controlled day and night for 12 h:12 h. They had ad libitum access to food and water. All samples were dissolved in sterile saline containing 5% Tween 80 immediately before use and were orally administered to mice (*n* = 8 per group) at 17:00 h. The experimental procedure and timeline for analyzing sleep architecture are depicted in Fig. 1. Mice were treated as follows: on day 0, they received the vehicle to obtain baseline (BL) data; for the next 21 days, they were administered either RBS or ZPD; and during the last 2 days (withdrawal period, WD), they were given the vehicle again (WD1 and WD2).

### Polysomnographic Recordings and Analysis of Vigilance States

Under pentobarbital anesthesia (50 mg/kg, i.p.), C57BL/6N mice were surgically implanted with a headmount (#8201, Pinnacle Technology Inc., USA) equipped with electroencephalograms (EEG) and electromyograms (EMG) electrodes for polysomnographic recordings. The front edge of the headmount was positioned 3.0 mm anterior to bregma, and four EEG electrode screws were placed in pre-drilled holes in the skull. Two EMG wire electrodes were inserted into the nuchal muscles. The headmount was secured to the skull with dental cement. After surgery, mice were given 1 week to recover in individual cages, followed by 3–4 days of habituation to the recording conditions before the experiment. EEG and EMG recordings were conducted using a slip ring system that allowed unrestricted movement. Recordings were taken during BL, on days 1, 7, 14, and 21, and during the withdrawal period using the PAL-8200 data acquisition system (Pinnacle Technology Inc.). The EEG and EMG signals were amplified (100×), filtered (EEG: low-pass filter at 25 Hz; EMG: low-pass filter at 100 Hz), and recorded at a sampling rate of 200 Hz. Mice were considered asleep when no EMG signal was detected. Vigilance states were automatically classified into Wake, REMS, or NREMS based on 10-sec epochs using SleepSign ver. 3.0 (Kissei Comtec, Japan), and final classifications were visually inspected and corrected if needed. Sleep latency was defined as the time from drug administration to the first NREMS episode lasting at least 120 sec [14].

### Statistical Analysis

All data are presented as the mean ± SEM. Statistical analyses were conducted using Prism 10.0 (GraphPad Software Inc., USA). Paired Student’s *t*-tests were used to analyze before and after comparisons. A significance level of *p* < 0.05 was applied for all statistical tests.

## Results

### Effects of Chronic Administration of RBS and ZPD on Sleep Latency, Amount of NREMS and REMS, and Time-Course Changes in NREMS

**Fig. 2:**
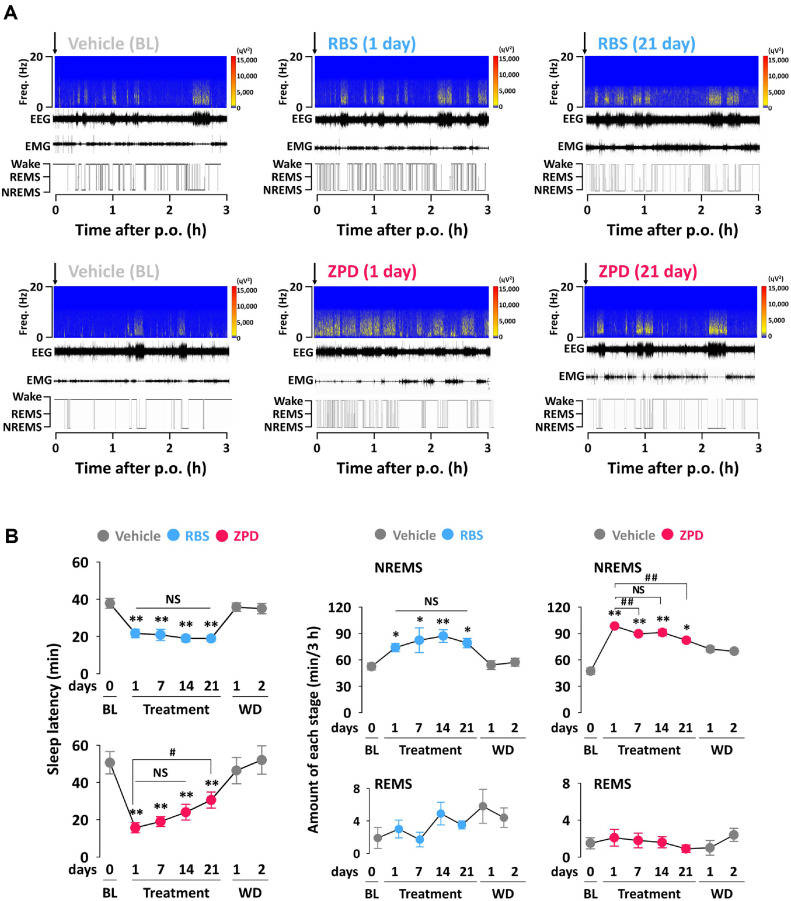
(A) Representative example of EEG (power spectrogram and wave trace), EMG, and corresponding hypnograms from a single mouse during the first 3 h after treatment with vehicle, RBS, and ZPD. (B) Effects of the chronic administration of RBS or ZPD on sleep latency and amounts of NREMS and REMS in C57BL/6N mice. Each value represents the mean ± SEM of each group (*n* = 7‒8). The data are expressed relative to recording days for the BL, treatment (1, 7, 14, and 21 days) and drug withdrawal (WD 1 and WD 2) days. *p < 0.05, **p < 0.01, significantly different from the BL (paired Student’s *t*-test). ##*p* < 0.01, significantly different from 1 days (paired Student’s *t*-test). BL, baseline; NS, no significant difference from 1 days (paired Student’s *t*-test); RBS, rice bran supplement; REMS, rapid eye movement sleep; NREMS, non-REMS; Wake, wakefulness; WD, withdrawal; ZPD, zolpidem.

Fig. 2A shows a representative example of EEG (power spectrogram and wave trace), EMG, and corresponding hypnograms from a single mouse during the first 3 h after treatment with vehicle, RBS, and ZPD. As shown in Fig. 2B, this study examined the effects of chronic administration of RBS (1,000 mg/kg) or ZPD (5 mg/kg) on sleep latency in mice. RBS significantly reduced sleep latency, with effects observed on day 1 (21.7 ± 2.3 min) and persisting until day 21 (18.9 ± 1.4 min) of the chronic administration period, compared to BL (37.8 ± 2.6 min). ZPD also significantly (*p* < 0.01) reduced sleep latency on day 1 (15.7 ± 2.7 min) compared to BL (55.6 ± 6.0 min). However, its effect progressively diminished with continued administration, and as a result, the sleep latency on day 21 was 30.6 ± 4.3 min. Sleep latency for both RBS and ZPD returned to baseline during the WD.

We next calculated the total time spent in NREMS and REMS during the 3 h following repeated administrations of RBS or ZPD (Fig. 2B). RBS increased the total NREMS amount by 1.4-fold and 1.5-fold on the first and last day compared to BL. Similarly, ZPD increased the amount of NREMS by 2.08-fold on day 1 compared to BL, and this effect persisted throughout the administration period on 21 days (1.74-fold). However, this effect gradually decreased during the ZPD administration period. No significant changes were observed in REMS amount following repeated administration of either RBS or ZPD.

**Fig. 3:**
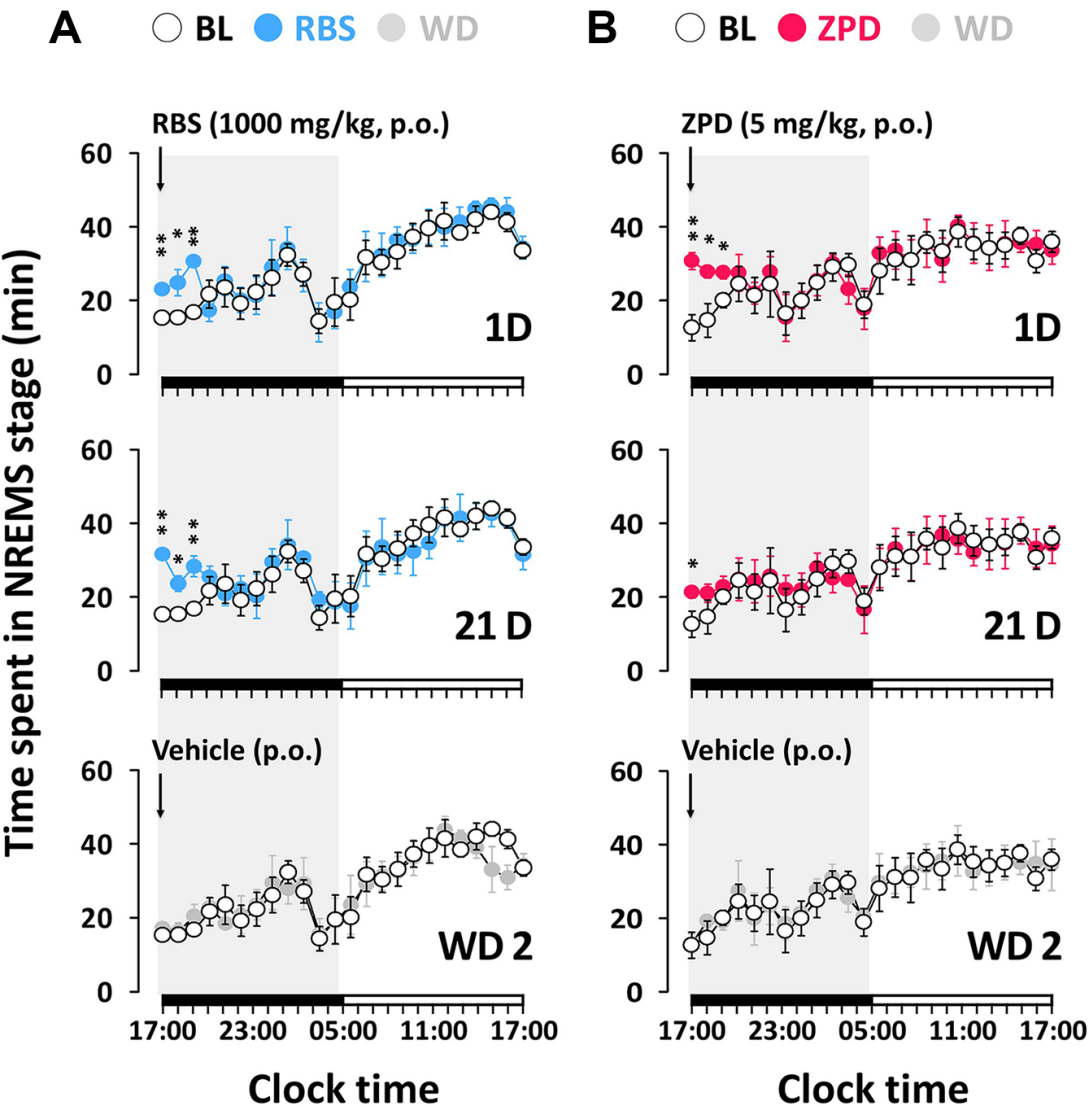
Effects of the chronic administration of RBS (A) and ZPD (B) on time-course changes in NREMS during the 24 h. Open and filled circles indicate the baseline day (vehicle) and experimental day (RBS, ZPD or WD), respectively. The data are expressed relative to recording days for the BL, treatment (1 D, 21 D) and drug withdrawal (WD 2) days. Each circle represents the hourly mean ± SEM (*n* = 7‒8) of NREMS. *p < 0.05, **p < 0.01, significantly different from the vehicle (paired Student’s *t*-test). The horizontal filled and open bars on the X-axis (clock time) indicate the 12 h dark and 12 h light periods, respectively. BL, baseline; NREMS, non- rapid eye movement sleep; RBS, rice bran supplement; WD 2, second day of withdrawal; 1 D, first day of treatment; 21 D, 21 days of treatment; ZPD, zolpidem.

Figure 3 shows the time course of in NREMS, REMS, and Wake during the 24 h period following RBS and ZPD administration on days 1, 21, and WD2. RBS significantly increased the amount of NREMS recorded during the first 3 h on both the day 1 and day 21 in mice, with no further effects on sleep architecture during the subsequent observation period. On the other hand, the hypnotic agent ZPD significantly increased the amount of NREMS on day 1 during the first 3 h. However, after 3 weeks of ZPD administration, the increase in NREMS was limited to just 1 h. During the WD, both RBS and ZPD maintained normal sleep architecture without significant dependence effects.

### Effect of Chronic Administration of RBS and ZPD on Sleep-Wake Episode Characteristics and Delta Activity

**Fig. 4:**
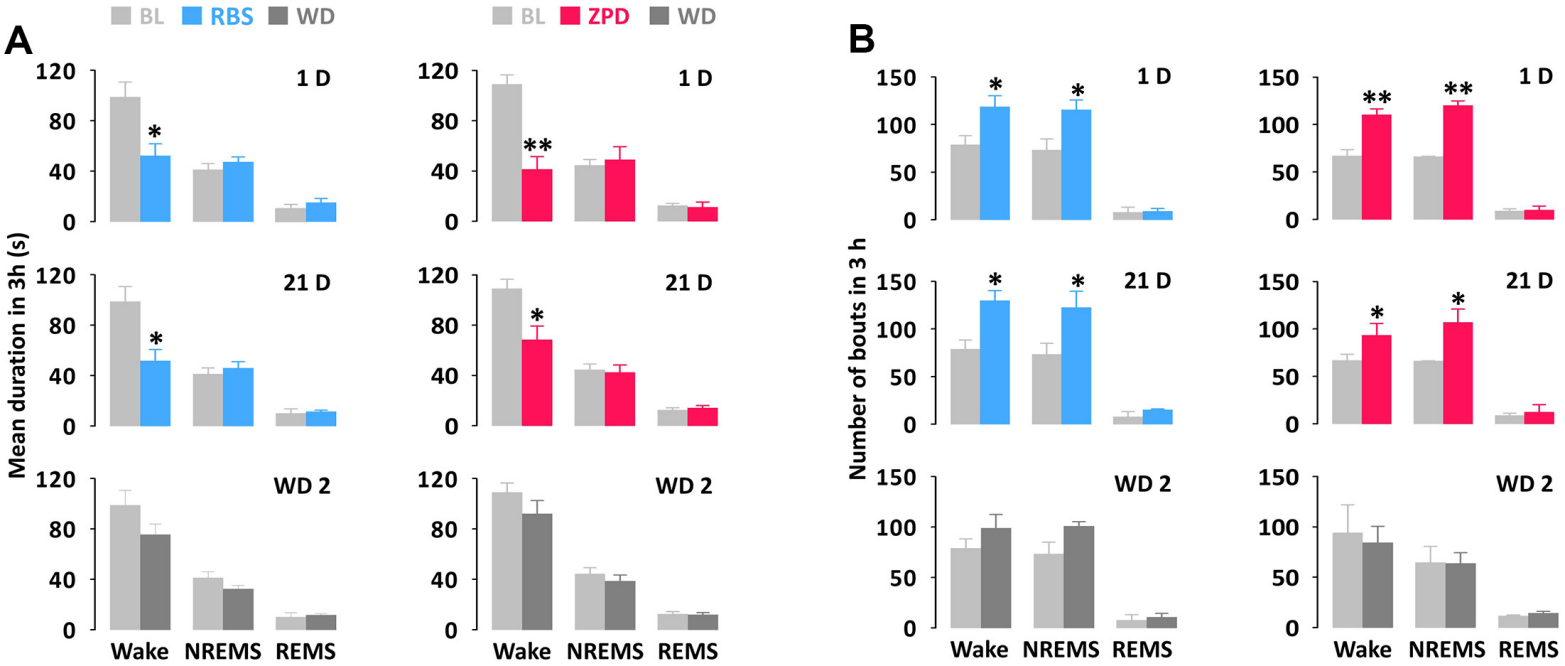
Characteristics of sleep-wake bouts in mice caused by chronic administration of RBS and ZPD. (A) Changes in mean duration of Wake, NREMS and REMS bouts. (B) Changes in total number of Wake, NREMS and REMS bouts. The data are expressed relative to recording days for the BL, treatment (1 D, 21 D) and drug withdrawal (WD 2) days. Each value represents the mean ± SEM of each group (*n* = 7‒8). *p < 0.05, **p < 0.01, significantly different from their vehicle (paired Student’s *t*-test). BL, baseline; RBS, rice bran supplement; REMS, rapid eye movement sleep; NREMS, non-REMS; Wake, wakefulness; WD 2, second day of withdrawal; 1 D, first day of treatment; 21 D, 21 days of treatment; ZPD, zolpidem.

To better understand the chronic administration effects of RBS and ZPD, the mean duration and total number of NREMS, REMS, and Wake episodes were performed (Fig. 4). RBS significantly reduced the mean duration of Wake on both day 1 and 21 day by 47.2 and 47.7%, compared to the BL. However, those of NREMS and REMS were not affected (Fig. 4A). Additionally, RBS significantly increased the number of NREMS bouts by 1.6- and 1.7-fold on the day 1 and day 21, respectively, and the number of Wake by 1.5- and 1.6-fold, respectively (*p* < 0.05, Fig. 4B). For ZPD, the mean duration of Wake mean duration on day 1 decreased by 61.9% compared to BL, but by day 21, the reduction was only 37.3% (Fig. 4A). Additionally, on day 1, the number of NREMS and Wake bouts increased by 1.81-fold and 1.65-fold, respectively, compared to BL. By day 21, these increases were 1.61-fold and 1.40-fold (Fig. 4B). This suggests that the sleep efficacy of ZPD diminished with prolonged oral administration. On the second day of WD, neither RBS nor ZPD affected the mean duration or number of bouts (Fig. 4A and 4B).

**Fig. 5:**
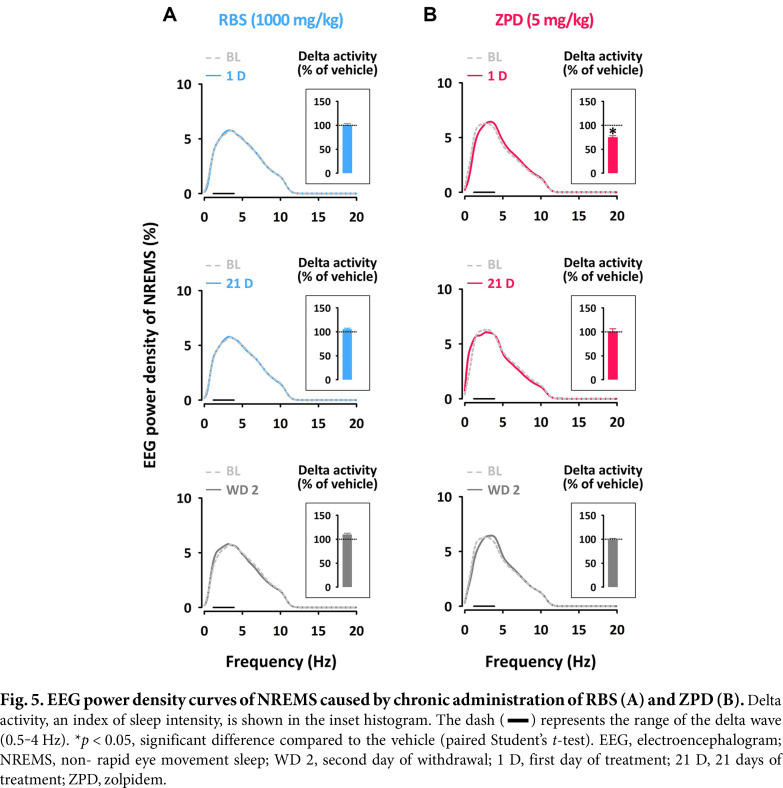
Fig. 5

As shown in Fig. 5A, there was no significant difference in the delta activity (0.5–4 Hz frequency range) in the EEG power density of NREMS between RBS treatment and the BL on day 1, and this lack of difference persisted throughout the entire treatment period. In contrast, ZPD reduced delta activity by approximately 25% on day 1. However, by day 21 and the second day of WD, delta activity in the ZPD group was similar to that of the BL (Fig. 5B).

## Discussion

Chronic administration of sleep drugs has been reported to not only reduce therapeutic efficacy but also cause withdrawal symptoms upon discontinuation [15]. Representative withdrawal symptoms include sleep disturbances, restlessness, anxiety, tremors, and dizziness. In contrast, natural sleep aids are generally considered to have fewer side effects than conventional synthetic sleep drugs [16]; however, apart from toxicity assessments, scientific evidence regarding their long-term use remains insufficient. In fact, benzodiazepine-like drugs such as ZPD are not recommended for continuous prescription beyond four weeks [17], and it has been reported that taking them for more than ten days may lead to side effects such as tolerance [18].

Sleep-functional foods fall under the over-the-counter category, which means they can be purchased without a prescription and are likely to be consumed continuously over the long term. Therefore, it is essential to evaluate the long-term efficacy and potential withdrawal effects of these products [19]. In the present study, repeated administration of ZPD for three weeks resulted in a gradual decrease in sleep-promoting effects from the first day of administration to day 21, whereas RBS maintained consistent sleep-promoting effects over the same period.

ZPD binds to the benzodiazepine (BZD) binding site located at the interface of the a and γ subunits of the GABA_A_ receptor. This binding enhances GABAergic neurotransmission by promoting chloride ion channel opening, thereby strengthening neural inhibition, lowering brain excitability, and inducing sleep [20]. However, GABA_A_-BZD receptor agonists such as ZPD and DZP develop tolerance with long-term administration: total sleep time increases during the first week but returns to baseline after two to three weeks, and during withdrawal, total sleep time decreases significantly [7, 21].

The development of tolerance is generally interpreted as a reduction in BZD binding sites [22]. In a study using HEK 293 cells expressing recombinant α_1_β_2_γ_2s_ receptors, short-term (2-h) exposure did not alter the [³H] flunitrazepam binding site, whereas long-term (48-h) exposure increased the number of binding sites and significantly decreased GABA’s ability to enhance binding [23]. Additionally, Marshall *et al*. (1997) [24] observed that chronic BZD administration reduced the mRNA expression of certain α subunits, suggesting that tolerance and dependence might be related to changes in receptor subunit composition, and in rodents, GABA_A_-BZD receptor agonists have been reported to fail in maintaining consistent effects with long-term administration [25, 26].

In our previous study, we confirmed that RBS promotes sleep by binding to the histamine H_1_ receptor (H_1_R)[12]. H_1_R plays a crucial role in the sleep-wake cycle [27], and its antagonists are effective in enhancing NREM sleep. For example, doxepin and diphenhydramine are widely used for the treatment of insomnia [28], and clinical studies have shown that long-term administration of doxepin does not result in next-day residual effects, memory impairment, or complex sleep behaviors [29, 30].

In a comparative study, Drake *et al*. (2017) [31] reported that while 10 mg of ZPD produced strong central nervous system (CNS) depressant effects impairing next-day alertness and balance, 6 mg of doxepin was associated with lower arousal thresholds and a reduced risk of falls. ZPD has been linked to sedative tolerance, withdrawal symptoms, and changes in GABA_A_ receptor subtypes following short-term use [32], whereas low-dose doxepin does not induce such neuroadaptive responses. BZD drugs act on the exogenous binding sites of the GABA_A_ receptor, and it has been reported that tolerance readily develops with long-term administration due to compositional changes and internalization [33].

In contrast, histamine receptors are continuously regulated by endogenous histamine, maintaining more stable pharmacological effects [34]. Therefore, since RBS has the same mechanism as doxepin while operating under the regulation of endogenous histamine, it is expected to pose a lower risk of tolerance and withdrawal symptoms compared to ZPD when administered long-term. Moreover, the fact that RBS is naturally derived is considered an important factor further supporting its safety profile.

In conclusion, ZPD showed a reduction in sleep-promoting efficacy with long-term administration in mice, while our study confirmed that RBS maintained consistent efficacy even after three weeks of repeated administration in mice. These findings suggest that RBS may provide a stable sleep-aid effect without inducing tolerance or withdrawal symptoms, offering valuable foundational data for evaluating the long-term efficacy of natural sleep aids. However, to fully validate these results, additional clinical trials on long-term intake, as well as mechanistic studies, are essential.
